# Physalin A exerts anti-tumor activity in non-small cell lung cancer cell lines by suppressing JAK/STAT3 signaling

**DOI:** 10.18632/oncotarget.7051

**Published:** 2016-01-28

**Authors:** Fanfan Zhu, Chunyan Dai, Yufei Fu, Jacky F.C. Loo, Dajin Xia, Sizhi P. Gao, Zhongjun Ma, Zhe Chen

**Affiliations:** ^1^ Zhejiang Key Laboratory of Gastro-Intestinal Pathophysiology, Zhejiang Hospital of Traditional Chinese Medicine, Zhejiang Chinese Medical University, Hangzhou, PR China; ^2^ Biochemistry Program, School of Life Sciences, The Chinese University of Hong Kong, Hong Kong; ^3^ Zhejiang University School of Public Health, Zijingang Campus, Hangzhou, PR China; ^4^ HOPP, Memorial Sloan Kettering Cancer Center, New York, NY, USA; ^5^ Institute of Marine Biology and Natural Products, Ocean College, Zhejiang University, Zijingang Campus, Hangzhou, PR China

**Keywords:** JAK/STAT3, non-small cell lung cancer, physalin A, withanolide

## Abstract

The signal transducers and activators of transcription 3 (STAT3) signaling pathway plays critical roles in the pathogenesis and progression of various human cancers, including non-small cell lung cancer (NSCLC). In this study, we aimed to evaluate the therapeutic potential of physalin A, a bioactive withanolide derived from Physalis alkekengi var. francheti used in traditional Chinese medicine, was evaluated in human NSCLC cells. Its and determined whether it effect oninhibited both constitutive and induced STAT3 activity, through repressing the phosphorylation levels of JAK2 and JAK3, resulting in anti-proliferation and pro-apoptotic effects on NSCLC cells was also determined, and. theThe antitumor effects of physalin A were also validated usingin an *in vivo* mouse xenograft models of NSCLC cells. Physalin A had anti-proliferative and pro-apoptotic effects in NSCLC cells with constitutively activated STAT3; it also suppressed both constitutive and induced STAT3 activity by modulating the phosphorylation of JAK2 and JAK3. Furthermore, physalin A abrogated the nuclear translocation and transcriptional activity of STAT3, thereby decreasing the expression levels of STAT3, its target genes, such as Bcl-2 and XIAP. Knockdown of STAT3 expression by small interfering RNA (siRNA) significantly enhanced the pro-apoptotic effects of physalin A in NSCLC cells. Moreover, physalin A significantly suppressed tumor xenograft growth. Thus, as an inhibitor of JAK2/3-STAT3 signaling, physalin A, has potent anti-tumor activities, which may facilitate the development of a therapeutic strategy for treating NSCLC.

## INTRODUCTION

Lung cancer is the leading cancer killer worldwide with non-small cell lung cancer (NSCLC) accounting for 80% of lung cancers [1]. Although recent progress has been made with the development of epidermal growth factor receptor tyrosine kinase inhibitors (EGFR-TKIs), such as gefitinib and erlotinib, they are only effective for a subset of patients with tyrosine kinase activating mutations750) [2–5]. However, most NSCLC patients do not benefit from the TKI treatment. Of those few who do, many will eventually develop resistance, resulting from a secondary EGFR mutation (e.g., T790M) or activation of other signaling pathways [6–12]. Thus, there is an urgent need for the development of novel, effective and safe therapeutic agents for the treatment of NSCLC.

Natural compounds derived from medicinal plants have played an important role in combating cancer. For example, *Physalis alkekengi* var. franchetii (Solanaceae) has been widely used in traditional Chinese medicine for the treatment of sore throat, cough, eczema, hepatitis, urinary problems and tumors [13]. We have previously demonstrated that physalin A, a major bioactive steroidal component of *P. alkekengi* var. franchetii, possesses anti-inflammatory activity by modifying IKKβ through a Michael addition reaction [14]. In addition, physalin A can activate mitochondrial apoptotic pathways through p53-Noxa-mediated ROS generation in human melanoma A375–S2 cells [15]. It also activates the death receptor-associated extrinsic apoptotic pathways via the upregulation of Fas expression [16]. However, the molecular mechanism underlying its anti-tumor activities has not been fully elucidated.

Constitutive activation of signal transducers and activators of transcription 3 (STAT3) plays a critical role in the tumorigenesis and progression of various human malignances [17–20]. Notably, persistently activated STAT3 was observed in approximately 50% of late-stage NSCLC tumors analyzed [21]. STAT3 activation is highly regulated by intracellular kinases, such as Janus kinases (JAKs) and Src, which are hyperactivated in a wide range of human cancers, including NSCLC [22–24]. Therefore, inhibition of STAT3 signaling has been suggested to be a promising therapeutic strategy for the treatment of this malignancy.

In this study, we investigated the effect of physalin A on the proliferation, apoptosis, and JAK/STAT3 signaling pathway in NSCLC cell lines. In addition, the anti-tumor activity of physalin A was tested in an *in vivo* xenograft model. Our results indicate that physalin A is a promising anti-cancer agent with potential clinical application in the treatment of NSCLC.

## RESULTS

### Physalin A inhibits cell viability in human NSCLC cells with constitutively activated STAT3

**Figure 1:**
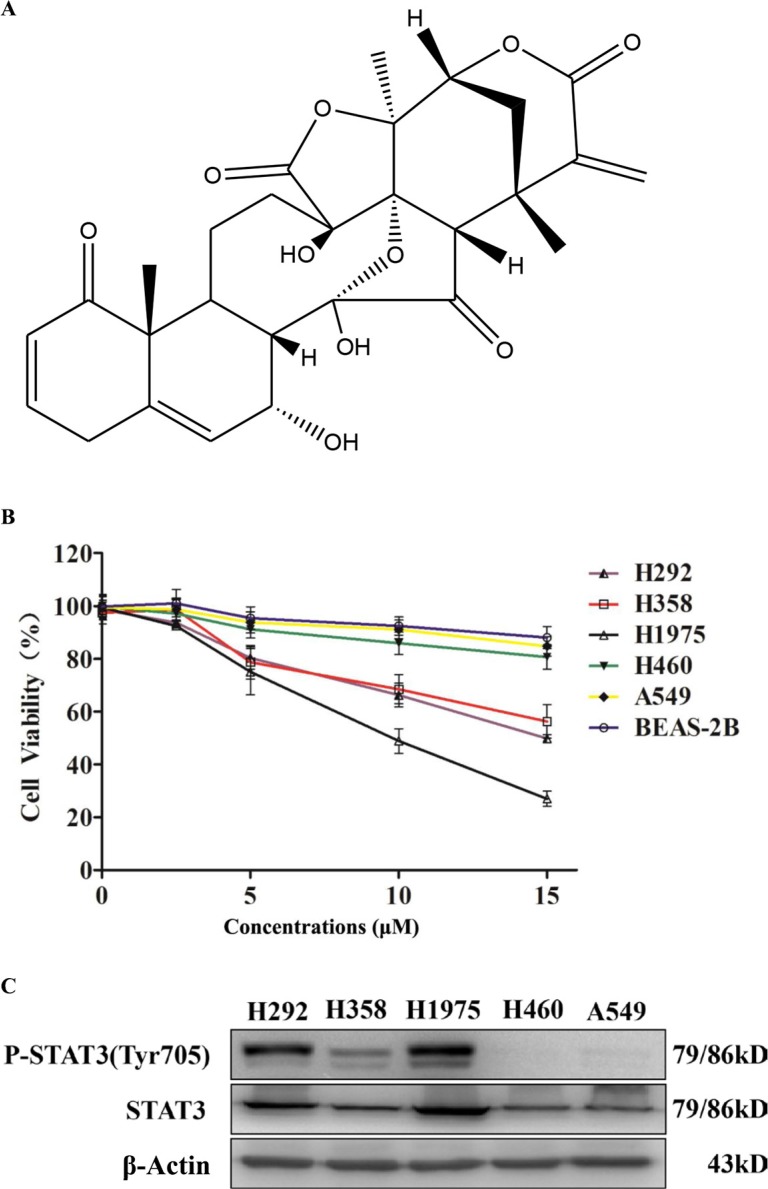
Physalin A exerts anti-proliferative effects in human NSCLC cells with activated STAT3 (**A**) Structure of physalin A. (**B**) The human NSCLC cell lines, H292, H358, H1975, H460, A549, and BEAS-2B (1 × 10^4^ cells/well) were treated with the indicated concentrations of physalin A for 24 h. Cell viability was then measured using the CCK-8 assay. Results are presented as mean ± SD from three independent experiments. (CB) p-STAT3 (Tyr 705) and STAT3 levels were detected in the H292, H358, H1975, H460 and A549 cell lines. β-actin was used as a loading control.

To determine the anti-proliferative effects of physalin A (structure shown in Figure 1A) in NSCLC cells, five human cell lines (H292, H358, H1975, H460, and A549 cells) were treated with various dosages of physalin A for 24 h. In addition, adenovirus-12 SV40 hybrid virus transformed, non-tumorigenic human bronchial epithelial (BEAS-2B) cells were also included as normal control epithelial cells. As shown in Figure 1B, physalin A at 15 μM slightly suppressed the viability of BEAS-2B cells by approximate 10–15%. Similarly, H460 and A549 cells were relatively resistant to physalin A. Compared to BEAS-2B, H460 and A549 cells, H292, H358 and H1975 cells at 5, 10, and 15 μM of physalin A were significantly sensitive to the inhibitory effect of physalin A (all *p* ≤ 0.002). Interestingly, physalin A induced higher growth inhibition in TKI-resistant H1975 cells than in H292 and H358 cells (10 and 15 μM, *p* ≤ 0.005, Figure 1B).

The levels of phosphorylated STAT3 at Tyr705 (Tyr705-p-STAT3) and total protein were next examined in all five NSCLC cell lines. p-STAT3 levels were high in H292, H358 and H1975 cells (Figure 1C), which were shown to be sensitive to physalin A (Figure 1B). In contrast, H460 and A549 cells, which were relatively resistant to physalin A, had almost undetectable levels of p-STAT3 (Figure 1C). Therefore, we hypothesized that the growth inhibitory effect of physalin A was mediated through its repression on STAT3 activation.

### Physalin A induces apoptosis of human NSCLC cells

**Figure 2:**
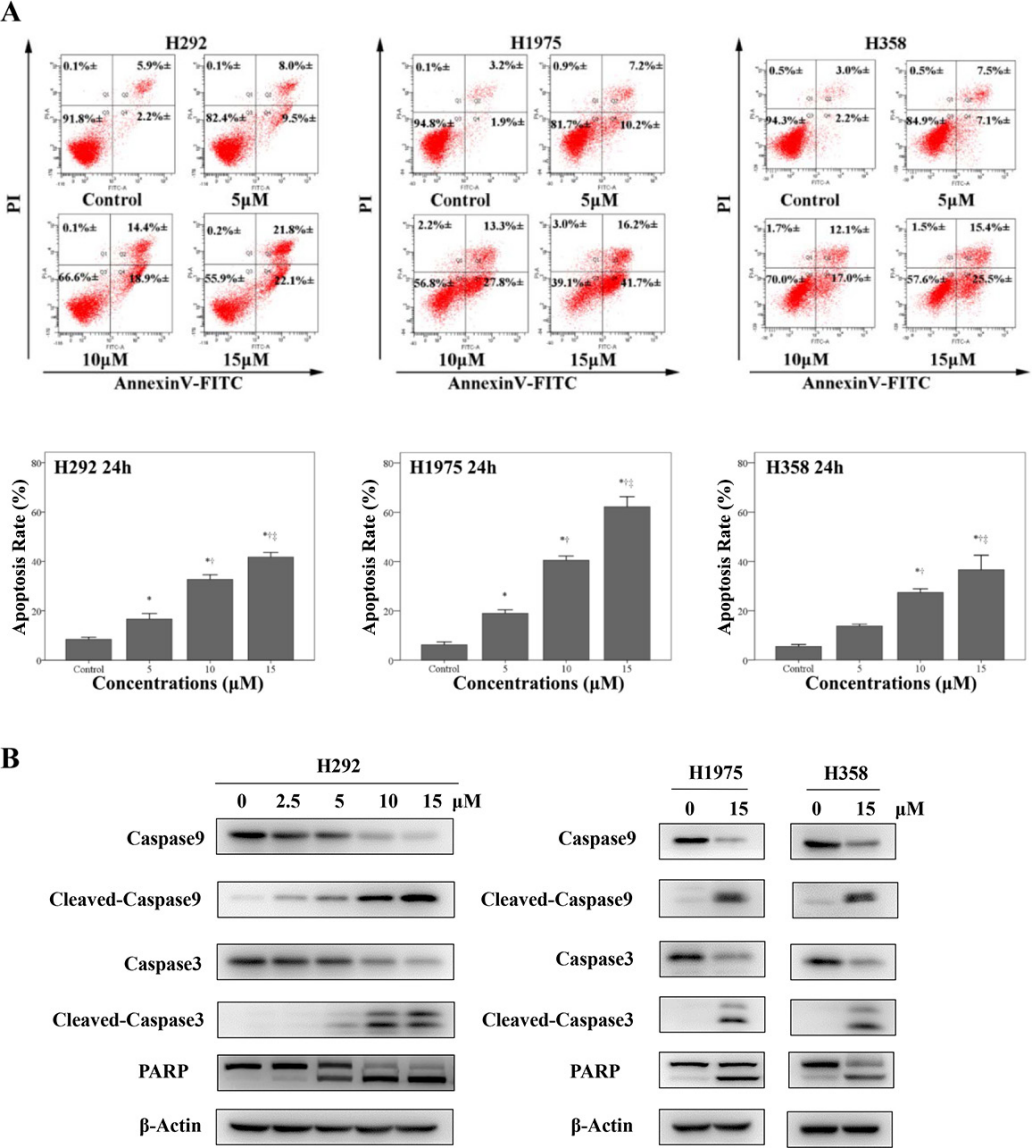
Physalin A induces apoptosis in human NSCLC cell lines (**A**) H292, H358 and H1975 cells (1 × 10^5^ cells/well) were treated with various concentrations of physalin A (PA) for 24 h, labeled with Annexin V-FITC/PI, and submitted to flow cytometric analysis. Numbers in the selected region represent the percentage of cells with the indicated fluorescence range in the total population. (**B**) H292 cells were treated with the indicated concentration of physalin A for 24 h, and H358 and H1975 cells were treated with 15 μM physalin A for 24 h. The expression of the apoptotic markers, cleaved caspase-9, cleaved caspase-3 and PARP, in addition to total Caspase-9 and Caspase-3 was determined by Western blot analysis. β-actin was used as a loading control. ^*,†,‡^*p* < 0.05, significantly different from the *control, ^†^5 μM physalin A-treated cells, and ^‡^10 μM physalin A-treated cells.

We next determined whether physalin A induced apoptosis of H292, H358 and H1975 cells. By examining Annexin V-FITC/PI flow cytometry, we found physalin A significantly increased apoptosis in H292, H358, and H1975 cells in a dose-dependent manner (at 15 μM physalin A, apoptosis rate: 41.7%, 62.2%, 36.6% for H292, H1975 and H358 cells, respectively, Figure 2A). Accordingly, physalin A substantially increased the levels of apoptotic markers, such as cleaved poly(ADP-ribose) polymerase (PARP), cleaved caspase-3 and cleaved caspase-9, in H292, H358, and H1975 cells (Figure 2B). However, total levels of caspase-3 and caspase-9 were reduced by physalin A (Figure 2B). These data suggest that physalin A induced apoptosis via repressing STAT3 activation, resulting in lowered NSCLC cell viability.

### Physalin A inhibits constitutive and IL 6-induced STAT3 phosphorylation at Tyr705

**Figure 3:**
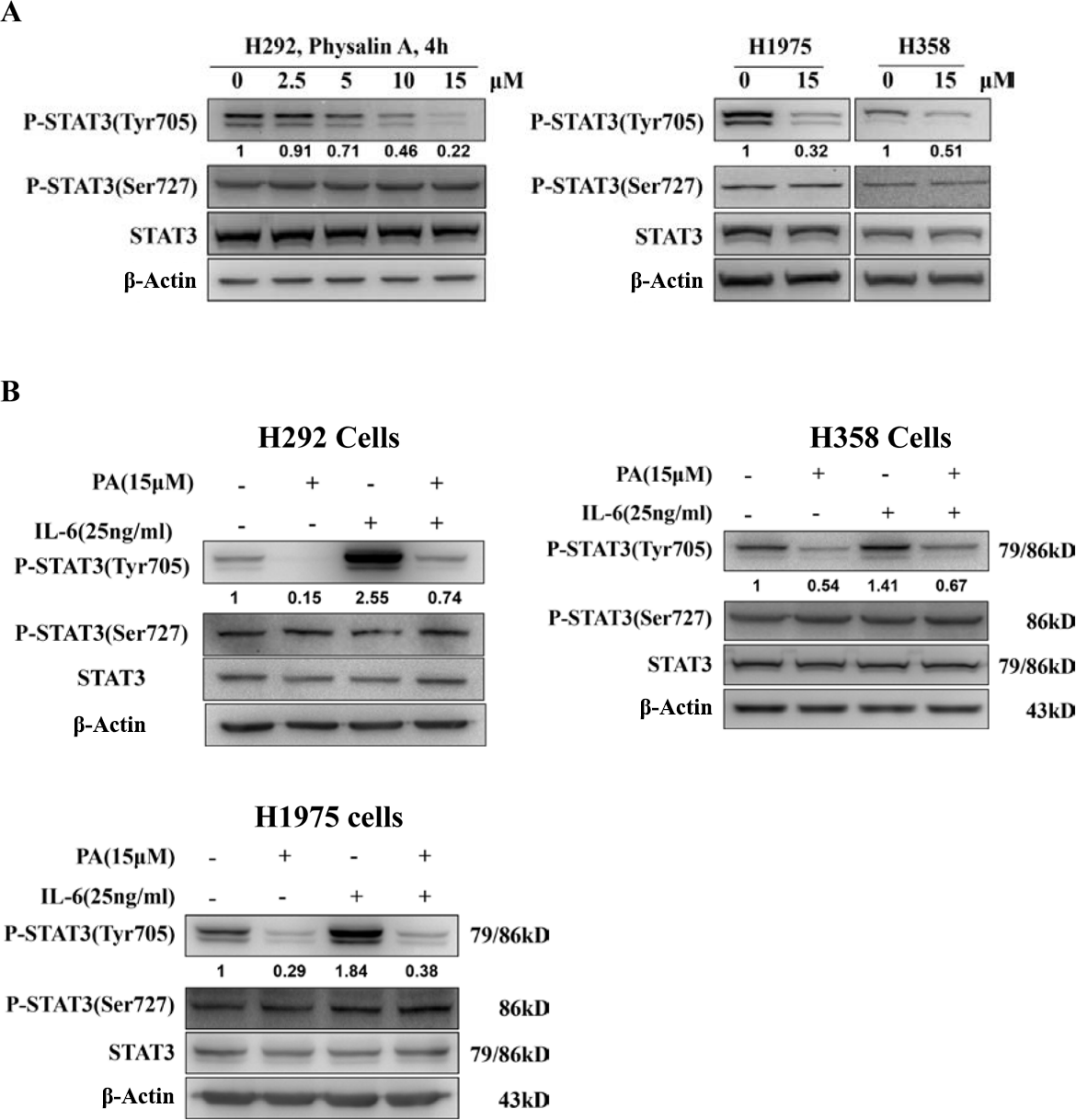
Physalin A inhibits constitutive and IL 6-induced STAT3 phosphorylation at Tyr705 (**A**) Physalin A (PA) inhibits STAT3 phosphorylation at tyrosine 705 but not at serine 727 in a dose-dependent manner. Cells were treated with the indicated concentration of PA for 4 h, and p-STAT3 (Tyr 705), p-STAT3 (Ser 727) and STAT3 levels were determined by Western blot analysis. β-actin was used as an internal loading control. (**B**) Cells were pretreated with PA for 6 h followed by 25 ng/mL of IL-6 to induce STAT3 tyrosine 705 phosphorylation. Cell lysates were subjected to Western blot analysis using antibodies specific for p-STAT3 (Tyr 705), p-STAT3 (Ser727), p-STAT3, STAT3 and β-actin.

Tyr705 and Ser727 are the critical phosphorylation sites for the activity of STAT3. To investigate whether physalin A suppressed STAT3 activity, H292 cells with considerable basal levels of activated STAT3 were treated with various concentrations of physalin A for 4 h. As shown in Figure 3A, physalin A inhibited Tyr705 phosphorylation of STAT3 in a concentration-dependent manner, while the basal Ser727 phosphorylation levels of STAT3 were not altered. Similarly, in H358 and H1975 cells, physalin A attenuated the levels of Tyr705-p-STAT3, but not Ser727-p-STAT3 (Figure 3A).

STAT3 is phosphorylated at tyrosine 705 by upstream kinases in response to cytokines and growth factors, including interleukin 6 (IL-6), oncostatin M, leukemia inhibitory factor (LIF) and epidermal growth factor (EGF) [25]. Therefore, we next determined whether physalin A could inhibit IL-6-induced STAT3 phosphorylation in IL-6 stimulated cells. As shown in Figure 3B, IL-6-induced Tyr705-p-STAT3 was substantially suppressed in NSCLC H292, H358 and H1975 cells. Thus, physalin A inhibited constitutive and IL-6-induced STAT3 Tyr705 phosphorylation in NSCLC cells.

### Small interfering RNA (siRNA) knock-down of STAT3 enhances physalin A-induced apoptosis

**Figure 4:**
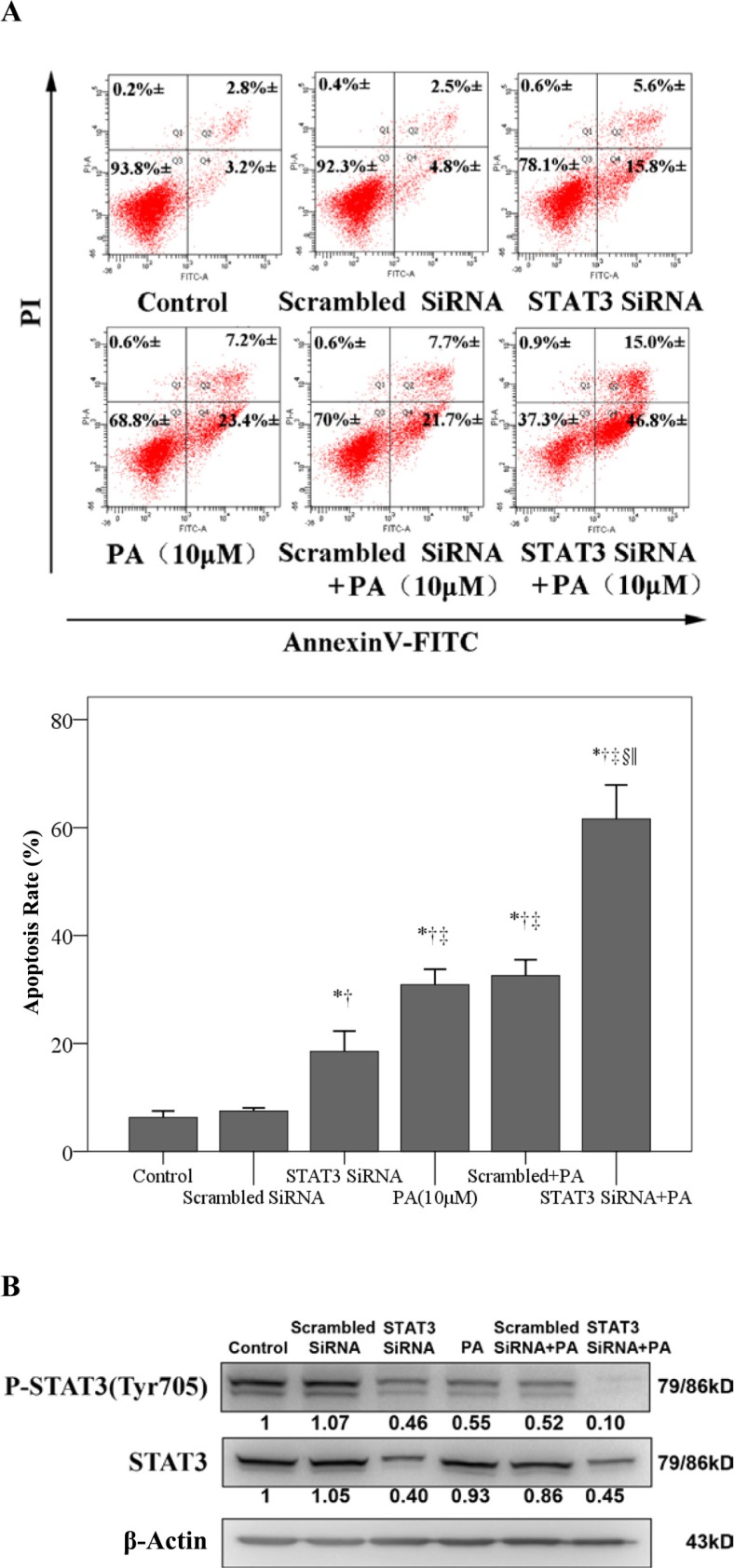
STAT3 siRNA enhances physalin A-induced apoptosis (**A**) H292 cells were treated with scrambled siRNA, STAT3 siRNA, 10 μM physalin A (PA), STAT3 siRNA+ 10 μM PA or scrambled siRNA+ 10 μM PA. Cell apoptosis was determined by flow cytometry using an Annexin V-FITC/PI staining kit. (**B**) Cell lysates were isolated for Western blot analysis to detect p-STAT3 (Tyr 705) and STAT3. β-actin was used as an internal loading control. ^*, †, ‡, §, ||^*p* < 0.05, significantly different from the *control, ^†^scrambled siRNA, ^‡^STAT3 siRNA, ^§^10 μM PA and ^||^STAT3 siRNA^+^ 10 μM PA groups.

We next investigated whether the siRNA suppression of STAT3 expression enhanced the pro-apoptotic effect of physalin A. As shown in Figure 4B, siRNA reduced STAT3 protein levels by approximately 60%, accompanied by an 18.5% increase of apoptosis. More interestingly, the combination of physalin A treatment with siRNA significantly enhanced cell apoptosis (61.6% vs. 30.9% 10 μM physalin A alone, *p* < 0.001, Figure 4A).

Analysis of total and phosphorylated STAT3 levels revealed that whereas STAT3 siRNA or physalin A alone induced a moderate reduction of p-STAT3 levels, the combination of siRNA and physalin A completely abrogated the Tyr705-p-STAT3 expression in H292 cells (Figure 4B). Therefore, siRNA-mediated knockdown of STAT3 expression significantly enhanced the pro-apoptotic effects of physalin A, supporting the critical role of STAT3 inhibition in the induction of apoptosis by physalin A.

### Physalin A suppresses STAT3 nuclear translocation and transcriptional activity

**Figure 5:**
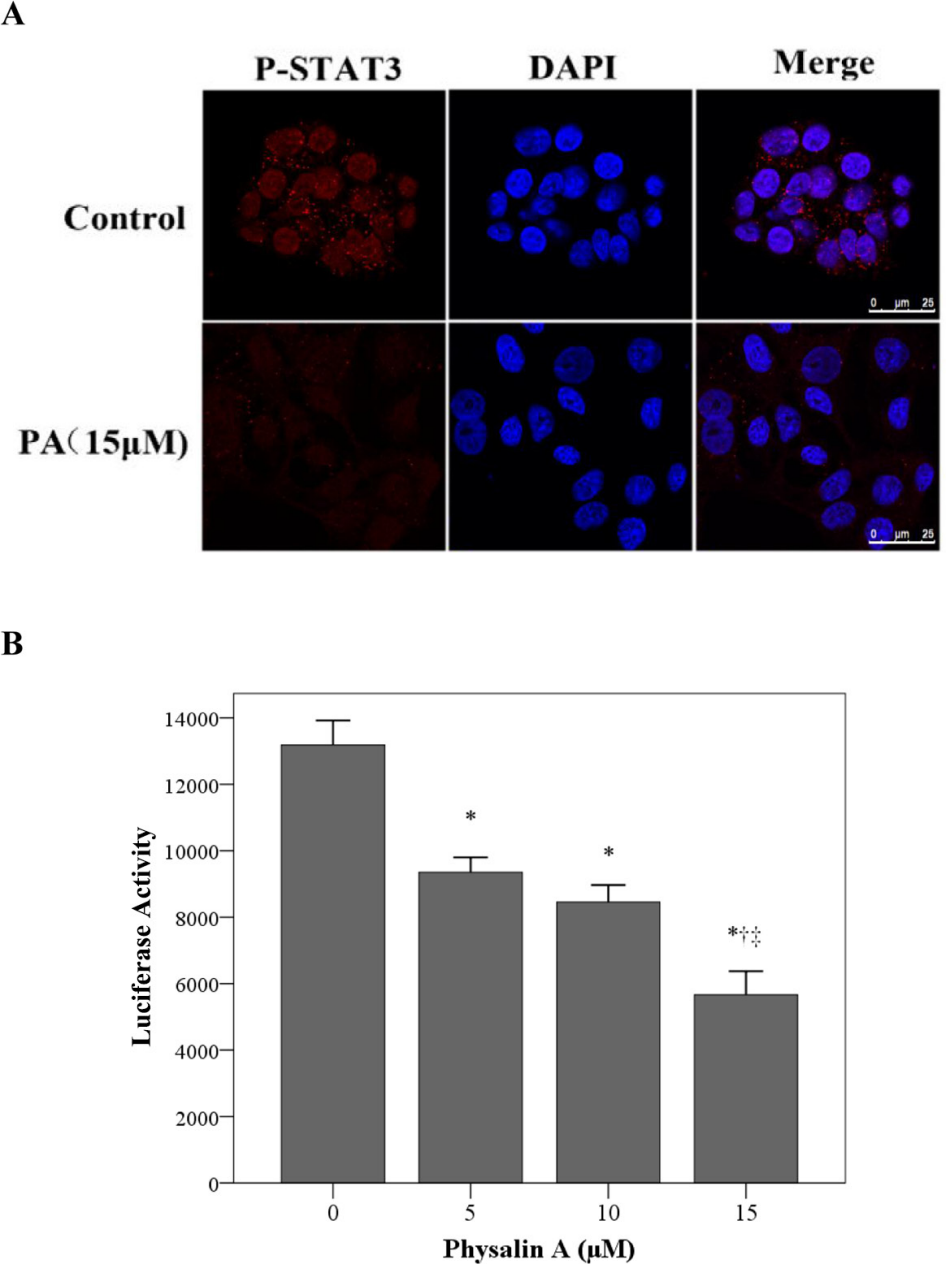
Physalin A suppresses p-STAT3 nuclear translocation and STAT3-dependent luciferase activity (**A**) H292 cells were treated with 15 μM physalin A (PA) for 4 h. Immunofluorescence analysis was performed with a rabbit anti-p-STAT3 (Tyr 705) antibody followed by an anti-rabbit IgG Fab2 Alexa Fluor 555. Nuclei were stained with DAPI. Merge image showed the overlay of red Alexa Fluor 555 and blue DAPI fluorescence. (**B**) H292 cells were co-transfected with a STAT3-luciferase plasmid and a *Renilla* luciferase plasmid, and after 24 h, the cells were treated with 15 μM PA for 8 h. Cell lysates were analyzed for luciferase activity. Results are mean ± SD from three independent experiments. ^*, †, ‡^*p* < 0.05, significantly different from *untreated control cells and those treated with ^†^5 μM or ^‡^10 μM PA.

STyr705-phosphorylated STAT3 translocates from the cytosol to the nucleus as a homodimer and subsequently activates the transcription of downstream target genes. Thus, we next examined if physalin A could inhibit the nuclear translocation of p-STAT3 using immunofluorescence assay. After serum starvation for 12 h, H292 cells were incubated with 15 μM physalin A for 6 h after which immunofluorescence analysis was undertaken. As shown in Figure 5A, Tyr705-p-STAT3 was predominantly localized in the nucleus of control H292 cells. In contrast, the nuclear level of p-STAT3 was dramatically decreased by physalin A.

To further confirm the inhibitory effect of physalin A on STAT3 transcriptional activity, we evaluated STAT3-dependent transcriptional activation using dual-luciferase reporter assays. As shown in Figure 5B, physalin A significantly decreased STAT3-specific luciferase activity in H292 cells.

**Figure 6:**
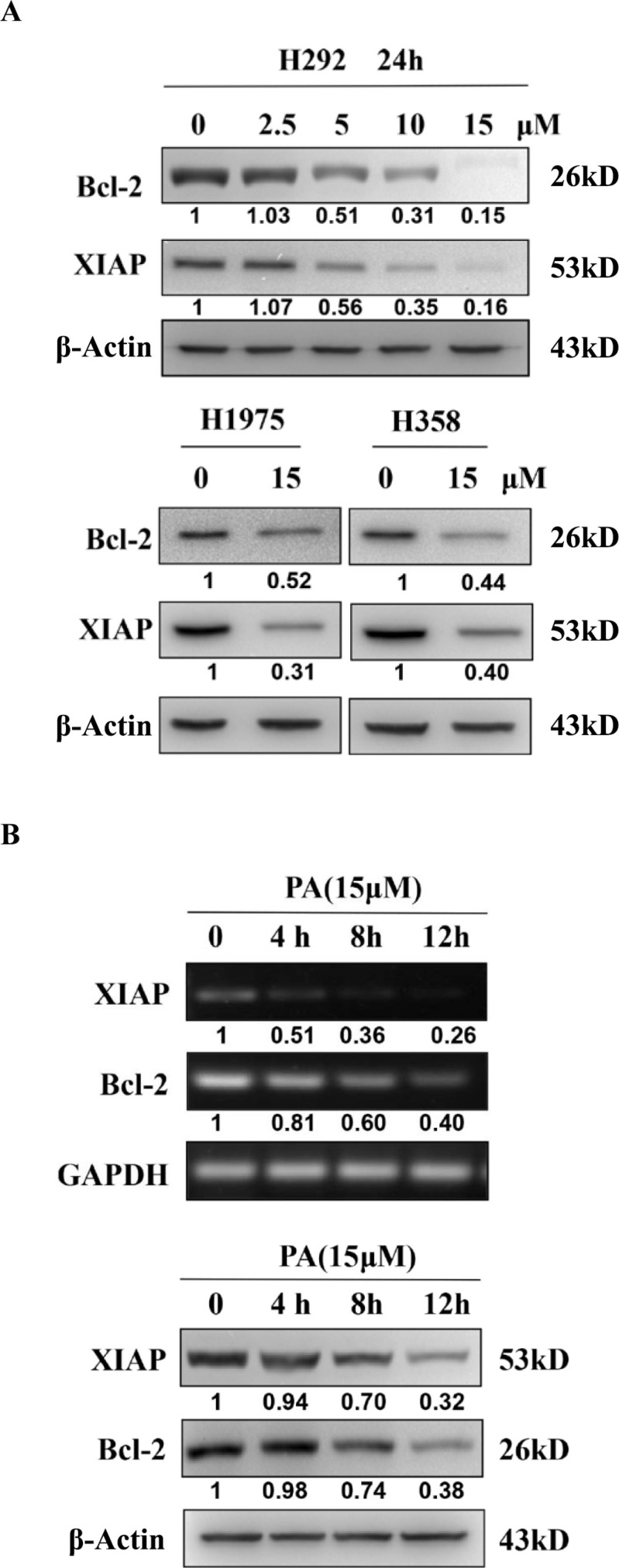
Physalin A suppresses the expression of the STAT3 target genes, Bcl-2 and XIAP (**A**) H292, H1975, and H358 cells were incubated with various concentrations of physalin A (PA) for 24 h. Cell lysates were isolated for Western blot analysis to detect Bcl-2 and XIAP protein levels. β-actin was used as a loading control. (**B**) H292 cells were treated with 15 μM PA for 4, 8 and 12 h after which the mRNA and protein levels of Bcl-2, XIAP and GAPDH or β-actin were detected by RT-PCR and Western blot analyses, respectively. GAPDH was used as a loading control for RT-PCR, and β-actin was used as a loading control for Western blot analysis.

Next, to confirm that physalin A suppressed STAT3-mediated gene expression and elucidated the mechanism underlying its pro-apoptotic effects, we analyzed the expression levels of STAT3 downstream target genes, *Bcl-2* and *XIAP*. As shown in Figure 6, the mRNA and protein levels of Bcl-2 and XIAP were significantly decreased in H292, H1975 and H358 cells by physalin A. Therefore, physalin A suppressed STAT3 transcriptional activity, resulting in down-regulation of its target genes, the anti-apoptotic Bcl-2 and XIAP.

### Physalin A inhibits phosphorylation of JAKs in NSCLC cells

**Figure 7:**
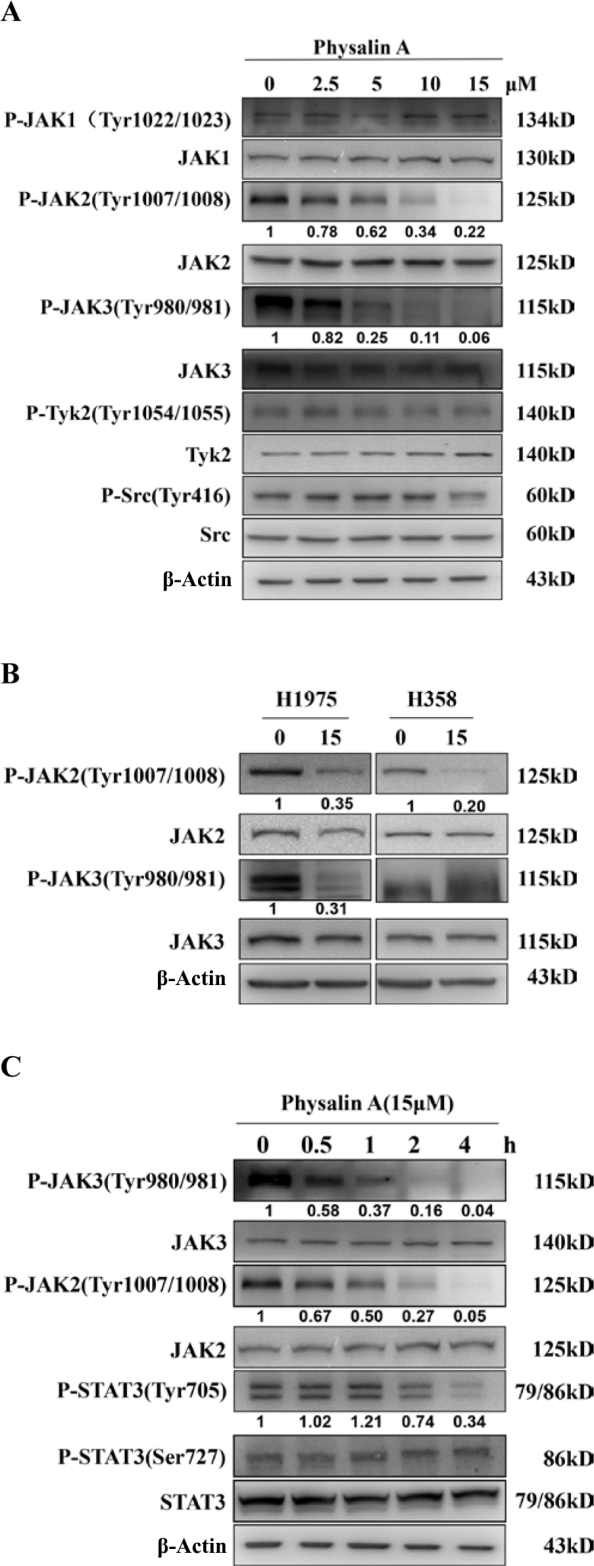
Physalin A inhibits phosphorylation of JAKs in human NSCLC cells in a dose- and time-dependent manner (**A**) H292 cells were incubated with the indicated concentrations of physalin A for 4 h, and the phosphorylation levels of the Janus family of tyrosine kinases (Jak1, Jak2, Jak3, and Tyk2) and Src were determined by Western blotting. (**B**) H1975 and H358 cells were incubated with 15 μM physalin A for 4 h, and the phosphorylation levels of Jak2 and Jak3 were determined by Western blotting. (**C**) H292 cells were incubated with 15 μM physalin A for 0.5, 1, 2 and 4 h, and Western blotting was performed to determine the time-dependent phosphorylation level of Jak1, Jak2, Jak3, Tyk2 and SRC and STAT3. β-actin was used as a loading control.

Tyrosine 705 phosphorylation of STAT3 can be induced by tyrosine kinases of the Janus family (JAKs) and Src kinases [22–24, 26]. To investigate whether physalin A inhibited constitutive activation of various kinases in NSCLC cells, we treated H292 cells with physalin A for 4 h and analyzed activation (phosphorylation) levels of the relevant tyrosine kinases. As shown in Figure 7A, physalin A inhibited the tyrosine phosphorylation of JAK2 and JAK3 in a dose-dependent manner, but did not affect that of JAK1 and tyrosine kinase 2 (TYK2). Similarly, physalin A reduced the levels of phosphorylated JAK2 (P-JAK2) in both H1975 and H358 cells, whereas only JAK3 phosphorylation (P-JAK3) was decreased in H1975 cells (Figure 7B).

We next examined the time course of physalin A effects on tyrosine kinase activity in H292 cells. Interestingly, the levels of p-JAK2 and p-JAK3 decreased as early as 30 min and were almost completely abrogated after 2 h of drug treatment, which coincided with a decrease in Tyr705-p-STAT3. These results suggest that physalin A-induced inhibition of p-STAT3 was mediated by reduced JAK activity.

### Physalin A suppresses tumor growth in a human H292 xenograft model

**Figure 8:**
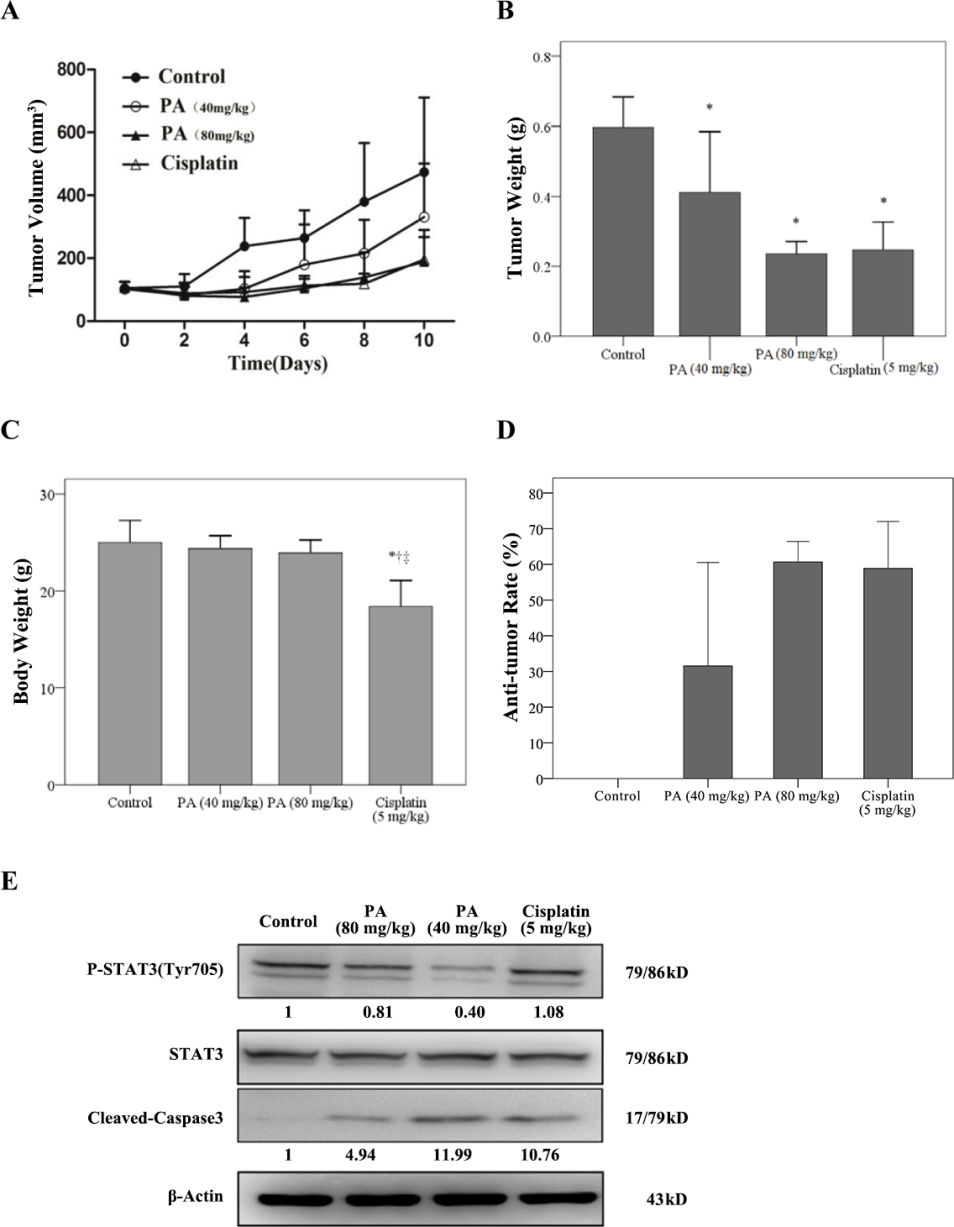
Physalin A suppresses tumor growth of H292 human NSCLC xenografts When subcutaneous H292 tumor xenografts reached 100–200 mm3, mice received PBS (1% DMSO and 5% Tween80), 40 mg/kg physalin A, 80 mg/kg physalin A, or 5 mg/kg cisplatin via intraperitoneal injection for 10 days. (**A**) Tumor volumes were measured at the indicated times using Vernier calipers. (**B**) The average tumor weight and (**C**) body weight of each group was measured on the last day of the experiment (day 10). (**D**) The anti-tumor rate was also determined at the end of the experiment. (**E**) Protein levels of pSTAT3 (Tyr705), total STAT3 and cleaved-caspase 3 in tumor tissues of mice in each treatment group. β-actin was used for normalization. ^*, †, ‡^*p* < 0.05, significantly different from the *control, ^†^PA (40 mg/kg), and ^‡^PA (80 mg/kg) groups.

The anti-tumor activity of physalin A *in vivo* was evaluated using NSCLC H292 xenograft models. Compared with the control mouse group, the mean tumor volume and tumor weight were significantly lower for the physalin A-treated groups (*p* ≤ 0.006; Figure 8A and 8B), with minimal impact on the mouse body weights (Figure 8C). The tumor inhibitory effect of physalin A was similar to cisplatin (Figure 8D). Analysis of the tumor samples revealed that physalin A decreased Tyr705-p-STAT3 levels, and increased caspase-3 activation (Figure 8E). Taken together, these results demonstrated that physalin A had anti-tumor activity that is comparative with the conventional chemotherapy agent, cisplatin, without the side effect of weight loss.

### Physalin A does not alter expression levels of protein tyrosine phosphatase (PTP), NF-κB activity, or suppressors of cytokine signaling (SOCS)

Many natural product-derived small molecules inhibit the phosphorylation of JAKs and STAT3 indirectly, via increasing the expression of PTPs and SOCSs [27–30]. In contrast, physalin A had minimal effects on the expression levels of PTPs, such as SHP-1, SHP-2 and PTEN (Supplementary Figure 1A). In addition, physalin A did not affect the protein levels of NF-κB and p-NF-κB (Supplementary Figure 1A) and nuclear translocation of p-NF-κB (Supplementary Figure 1B). Furthermore, the expression level of SOCS-1 and SOCS-3 was not affected by the treatment of physalin A in H292 cells (Supplementary Figure 1C). Therefore, the inhibition of JAK-STAT3 signaling by physalin A might be specifically JAK-dependent.

## DISCUSSION

In this study, we demonstrated that physalin A had potent anti-proliferative activity against NSCLC cells with constitutively activated STAT3, but less effect on the NSCLC cells with lower levels of activated STAT3, or non-transformed human lung epithelial cells. We next showed that physalin A inhibited JAK2/JAK3 activity, repressed the levels of activated STAT3, and downregulated the expression of STAT3 target genes, such as *Bcl-2* and *XIAP*, resulting in increased apoptosis. Lastly, we demonstrated that in xenograft NSCLC tumors, physalin A inhibited STAT3 Tyr705 phosphorylation and suppressed the *in vivo* tumor growth, to a degree comparable to cisplatin but with less detrimental influence to the body weight.

STAT3 activation requires phosphorylation of Tyr705, resulting in trans-modulation of downstream target genes that are involved in cell proliferation, survival, angiogenesis and metastasis [31–33]. In the present study, physalin A specifically inhibited both constitutive and IL6-induced Tyr705 phosphorylation of STAT3, with minimal impact on Ser727 and the levels of total STAT3 protein. Physalin A can also dampen the nuclear translocation and transcriptional activity of STAT3, resulting in reduced expression levels of the anti-apoptotic target genes, *Bcl-2* and *XIAP* [34], which may account for the physalin A-induced apoptosis in H292, H1975 and H358 cells. The degree of apoptosis induction by physalin A is similar to that of other withanolides, withaferin A, in uveal melanoma [35] and sativolide withanolides isolated from *Jaborosa reflexa* Phil. and *J. cabrerae* Barboza [36]. These results are also consistent with the findings of Hsu et al. [37], in which physalin B, a major constituent of the medicinal herb, *Physalis angulata* L., induced apoptosis in human melanoma A375 cells via increased expression levels of NOXA, Bax and caspase-3. In addition, physalin F, another *P. angulata* L. component, induced reactive oxygen species (ROS)-mediated apoptosis of human renal cancer A498 cells [38]. Furthermore, the current study demonstrated that physalin A displayed minimal cytotoxicity to non-transformed bronchial epithelial cells (BEAS-2B), suggesting a level of anticancer specificity.

Intracellular and receptor kinases, JAKs, EGFR and src, are hyper-activated in various cancers (including NSCLC), contributing to the phosphorylation of STAT3 at Tyr705 [33, 39, 40]. Of the JAK family kinases, JAK1, JAK2, JAK3 and TYK2 can all play a critical role in the activation of STAT3 [41, 42]. Here, we showed that physalin A inhibited the phosphorylation of JAK2 and JAK3 in human NSCLC cells with minimal impact on that of JAK1, TYK2 and Src, suggesting that the inhibition of Tyr705-p-STAT3 was mediated by JAK2 and JAK3 inhibition. Our hypothesis is supported by two recent studies that showed a regulatory role of JAK2 in STAT3 activation in NSCLC cells [43, 44]. In contrast, JAK1 has been suggested to activate STAT3 activity in lung cancer cell lines [45]. Therefore, the molecular mechanisms by which JAK members activate STAT3 in lung cancer remain to be investigated *in vitro* and *in vivo*.

SOCS 1 and SOCS 3 are negative regulators of JAK/STAT3 [29, 46–49]. Specifically, SHP-1, SHP-2 and PTEN dephosphorylate protein tyrosine kinases (PTKs), such as JAKs [50–52]. In addition, SOCS 1 and SOCS 3 inhibit JAK/STAT3 signaling through direct binding and suppression of the gp130 receptor and their associated JAKs [48, 49]. However, physalin A did not significantly influence PTP expression, SOCS levels, or p-NF-κB levels and translocation. These results are in contrast to that reported by Wu et al. [38], in which physalin F suppressed NF-κB activation in human renal A498 cells. This apparent contradiction may be due to cell type-dependent differences. Nonetheless, the inhibition of JAK-STAT3 signaling by physalin A might be specifically JAK-dependent.

Although our data suggest that the anti-tumor effects of physalin A are mediated by suppression of JAK/STAT3 signaling, it remains possible that additional mechanisms exist to account for its anti-tumor cytotoxicity, especially given the antioxidant activity reported for physalin D, another component of *P. alkekengi* [53]. Thus, further studies will evaluate the impact of physalin A on the oxidative state of cancer cells. Furthermore, our current study only focused on physalin A, but other active component physalins of *P. alkekengi* L. 5–14 have also been identified in *P. alkekengi* L. Therefore, it is possible that another physalin or a combination of physalins could exert more potent cytotoxicity against human NSCLC cells, which needs to be further explored [54, 55].

In summary, physalin A, a natural JAK2/JAK3 inhibitor, induces apoptosis of human NSCLC cells through inhibition of the JAK/STAT3 signaling pathway. These findings support the further development of physalin A as a potential anticancer drug for treating human NSCLC.

## MATERIALS AND METHODS

### Materials and reagents

Physalin A was isolated from the roots of traditional Chinese medicine calyx with the fruit of Franchet Ground cherry, and its molecular structure and purity was determined by ^1^H NMR (500 MHz, CDC13) and ^13^C NMR (125 MHz, CDC13) as described previously [14]. The compound was dissolved in DMSO at a stock concentration of 20 mM, and aliquots were stored at −20°C. Recombinant IL-6 was purchased from PeproTech (Rocky Hill, NJ, USA). Primary antibodies against cleaved PARP, caspase-3, caspase-9, cleaved-caspase-3, cleaved-caspase-9, Bcl-2, XIAP, p-STAT3 (Ser727), p-STAT3(Tyr705), STAT3, p-Jak1, p-Jak2, p-Jak3, p-Tyk2, Jak1, Jak2, Jak3, Tyk2, p-Src, Src, NF-κB, p-NF-κB (Ser536), SHP1, SHP2, PTEN, SOCS1, and SOCS3 were purchased from Cell Signaling Technology (Beverly, MA, USA). The primary antibody against p-Jak1 was purchased from Abcam (Cambridge, UK), and the primary antibody against β-actin was from Sigma-Aldrich (St. Louis, MO, USA). Goat anti-rabbit and goat anti-mouse secondary antibodies were purchased from Cell Signaling Technology. STAT3 small interfering RNA (sense: 5′-GGGACCUGGUGUGAAUUAUTT-3′, antisense: 5′-AUAAUUCACACCAGGUCCCTT-3′) and scrambled control siRNA (sense: 5′-UUCU CCGAACGUG UCACGUTT-3′, antisense: 5′-ACGUGACACGUUCGGA GAATT-3′) were purchased from Santa Cruz Biotechnology (Dallas, TX, USA). All absorbance and fluorescence data were obtained using a Varioskan^™^ Flash Multimode Reader (Thermo Fisher Scientific, Waltham, MA, USA).

### Cell culture and treatment

The human NSCLC cell lines, H292, H1975, H358, H460, A549 and non-cancerous bronchial epithelial cell line, BEAS-2B cells, were obtained from the Cell Bank of Type Culture Collection of Chinese Academy of Sciences (Shanghai, China). Cells were grown and maintained in RPMI 1640 (Life Technologies, Carlsbad, CA, USA) supplemented with 10% fetal bovine serum (Life Technologies) at 37°C with 5% CO_2_.

To determine if physalin A suppressed IL-6-induced STAT3 activation, H292, H358 and H1975 cells were serum-starved for 12 h, incubated with 15 μM physalin A for 6 h then exposed to 25 ng/mL of IL-6 for 15 min in the presence of physalin A.

### Proliferation assays

*In vitro* cytotoxicity was determined using the Cell Counting Kit-8 (CCK8; Sigma-Aldrich) assay, according to the manufacturer's protocol. Briefly, cells were seeded overnight on a 96-well plate (0.6 × 10^5^ cells per well) to achieve 70% confluence. Different concentrations of physalin A (0, 2.5 5, 10, and 15 μM) were added for 24 or 48 h after which 10 μL of CCK-8 solution was added to each well of the plate. After 2 h, the absorbance at 450 nm (OD450) was measured. *In vitro* cytotoxicity was assessed as the percentage of growth inhibition, which is calculated as 100% - [(OD450 of drug-treated cells/OD450 of untreated cells) × 100%].

### Annexin V assay

For flow cytometry analysis of annexin V externalization, cells were seeded overnight on a 12-well plate (1 × 10^5^ cells per well) to achieve 70% confluence. Different concentrations of physalin A (0, 5, 10, and 15 μM) were added for 24 h, and annexin V levels were determined using an Annexin V-FITC/PI staining kit (BD Pharmingen, San Diego, CA, USA) according to the manufacturer's protocol. Briefly, cells were trypsinized, washed twice with PBS and resuspended with Annexin V-FITC/PI binding buffer, and 1 × 10^5^ cells in 100 μL were incubated with anti-Annexin V-FITC and PI for 15 min at room temperature. After 400 μL of Annexin V-FITC/PI binding buffer was added, the cells were analyzed by flow cytometry using a BD FACSCanto II flow cytometry (BD Pharmingen) to detect the fluorescence signal.

### Western blot analysis

The cells were lysed with RIPA buffer (Beyotime, Shanghai, China) with a protease inhibitor cocktail (cOmplete ULTRA Tablets, Mini, EDTA-free, EASYpack, Roche, Basel, Switzerland), which contains serine, cysteine, and aspartic protease inhibitors, and the Phosphokinase Inhibitor Cocktail Set V (Merck KGaA, Darmstadt, Germany) containing the serine and cysteine protease inhibitors, 4- benzenesulfonyl fluoride hydrochloride, Aprotinin, E-64, and leupeptin hemisulfate. Protein concentrations were measured using a standard DC protein assay (Bio-Rad, Hercules, CA, USA), and 40 μg of protein was separated by SDS-PAGE and transferred to a PVDF membrane. After being blocked with 5% BSA for 1 h at room temperature, the membrane was incubated with primary antibodies diluted at 1:1000 overnight at 4°C, washed three times with TBST and incubated with secondary antibody for 1 h at room temperature. After being washed three times with TBST, the membranes were developed with New Clarity^™^ Western ECL Substrate (Bio-Rad), imaged and analyzed with a ChemiDoc^™^ XRS system and Quantity One software (Bio-Rad).

### STAT3 luciferase reporter assay

Luciferase assays were used to confirm p-STAT3 transactivation in the H292 cells, which were seeded on a 24-well plate at a density 1 × 10^5^ cells per well 24 h prior to transfection. The STAT3-TA-luc vector (kindly provided by HM Shen at the National University of Singapore) was mixed with *Renilla* luciferase vector (Promega, Fitchburg, WI, USA) at a 7:1 ratio. Lipofectamine 2000 (Life Technologies) was used according to the manufacturer's protocol, and physalin A was added 24 h post-transfection for an additional 8 h. Cells were harvested, and luciferase activities were determined using the Dual-Luciferase reporter assay (Promega).

### Immunocytochemical analysis of p-STAT3 localization

Immunofluorescence staining was used to confirm the localization of p-STAT3 in the cells. H292 cells were treated with 15 μM physalin A for 4 h, fixed in 4% cold paraformaldehyde for 15 min, and permeabilized with −20°C methanol for 10 min. After blocking with 5% BSA-0.3% triton-X for 1 h, the cells were incubated with anti-p-STAT3 antibodies diluted 1:100 overnight at 4°C followed by incubation with anti-mouse Alexa Fluor R555 (1:100; Life Technologies) for 1 h at room temperature. The nuclei were counterstained with DAPI.

### RT-PCR analysis

H292 cells were seeded in a 6-well plate (3×10^5^ cells per well) and cultured until they reached 70% confluence after which 15 μM physalin A was added and incubated for 4, 8 and 12 h. Total RNA was extracted with TRIzol (Life Technologies), and 1 μg of RNA was reverse transcribed using the iScript^™^ cDNA Synthesis Kit (Bio-Rad) with the primer sequences indicated in Table 1. All primers were synthesized from Sangon Biotech (Shanghai, China). PCR amplification conditions were as follows: 94°C for 2 min; 28 cycles of 94°C for 2 min, 55°C for 20 s, 72°C for 5 min; and 72°C for 5 min. All PCR products were visualized using 1% agarose gel electrophoresis and the ChemiDoc^™^ XRS system (Bio-Rad) for image capture and analysis.

### Xenograft mouse model

Xenograft tumors were established by subcutaneous injection of 5 × 10^6^ human H292 cells in 0.2 mL of PBS into the right front leg of BALB/c mice (mean age of 6 weeks). A total of 24 male BALB/c mice was used and divided into four groups (6 mice per group). When the tumor reached 100–150 mm^3^, PBS (1% DMSO and 5% Tween80), 40 mg/kg physalin A, 80 mg/kg physalin A, or 5 mg/kg cisplatin was injected intraperitoneally for 10 days. Tumor volumes were measured at 2-day intervals for the tumor growth curve. After 10 days, the mice were sacrificed, and tumors were removed and weighed. Protein extracts from the tumors were isolated to detect STAT3 and cleaved caspase-3 protein expression by Western Blot analysis. This study was carried out in accordance with local guidelines for the care of laboratory animals of Animal Experimental Center, Zhejiang Academy of Medical Sciences, and was approved by the ethics committee for research on laboratory animal use of the Zhejiang Chinese Medical University. Anti-tumor rates were defined as follows: the average tumor weight of the control group (g) - the average tumor weight of physalin A group (g)/the average tumor weight of the control group (g) × 100%.

### Statistical analysis

Comparisons between the groups or concentrations in cell viability, apoptosis rate, luciferase activity, tumor volume and weight, and body weight were performed by one-way ANOVA with Bonferroni post-hoc methods for multiple comparisons. Continuous values were presented as means and standard deviations (SDs). Statistic analyses were performed by IBM SPSS statistical software version 22 for Windows (IBM, Armond, NY, USA), and a two-tailed *P*-value < 0.05 indicated statistical significance.

## SUPPLEMENTARY MATERIALS FIGURE


