# Supplementary material for: Physalin A exerts anti-tumor activity in non-small cell lung cancer cell lines by suppressing JAK/STAT3 signaling
Source: Oncotarget. 2016 Jan 28;7(8):9462–76. doi: 10.18632/oncotarget.7051 (PMC4891052; doi:10.18632/oncotarget.7051)
Supplement: Supplementary file 1 [file oncotarget-07-9462-s001.pdf]

Physalin A exerts anti-tumor activity in non-small cell lung cancer cell lines by suppressing JAK/STAT3 signaling

Supplementary Materials

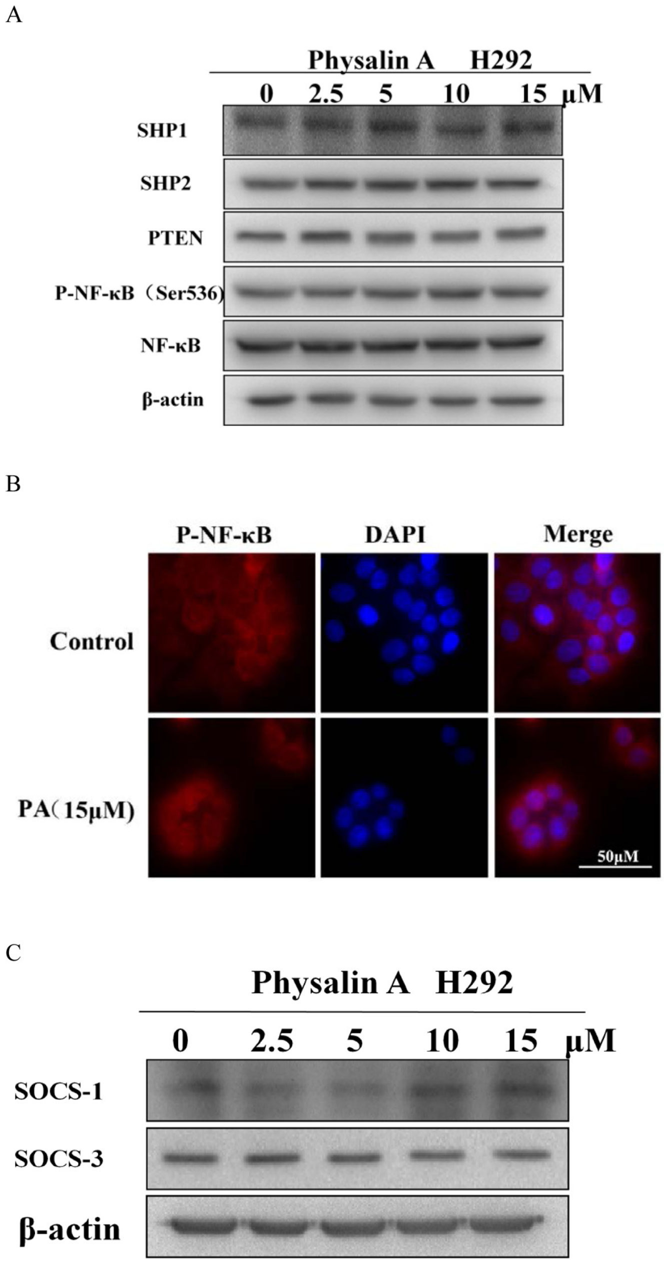

Supplementary Figure S1: Effects of Physalin A on protein expression of protein tyrosine phosphatases, NF-κB, and SOCS in H292 cells. (A, C) H292 cells were treated with the indicated concentrations of physalin A after which the protein expression of protein tyrosine phosphatases, NF-κB, p-NF-κB and SOCS was determined using Western blot analysis. (B) After H292 cells were treated with 15μM physalin A for 4 h, p-NF-κB subcellular localization was assessed by immunofluorescence analysis.
